# Effect of Pre‐Processing Treatment and Concentration of *Alaria esculenta*, *Saccharina latissima*, and *Laminaria digitata* Varieties on Texture and Consumer Attribute Preference of Crackers

**DOI:** 10.1002/fsn3.4710

**Published:** 2025-01-12

**Authors:** Moira Ledbetter, Katrina Ross, James Templeman, Boon‐Seang Chu, Jonathan D. Wilkin

**Affiliations:** ^1^ School of Applied Sciences, Division of Engineering and Food Science University of Abertay Dundee Scotland UK

**Keywords:** fortification, seaweed, temporal dominance of sensation (TDS), texture analysis, thermal treatments

## Abstract

This study investigates the effects of three brown seaweed species ( *Alaria esculenta* , *Saccharina latissima*, and *Laminaria digitata* ), their pre‐processing treatments, and incorporation percentages on the physical and sensory properties of crackers. Significant (*p* ≤ 0.001) two‐way and three‐way interactions were observed for moisture content, with seaweed addition generally resulting in drier crackers. Shrinkage was primarily influenced by sample thermal treatment, while hardness was significantly affected by seaweed species, treatment, and their interactions. The freeze–thaw treatment produced harder crackers compared to other treatments. Sensory analysis using temporal dominance of sensations (TDS) revealed variations in dominant attributes across different seaweed species and treatments, with retorted and freeze–thaw treatments enhancing crunchiness and reducing fishy flavors. The study demonstrates the potential of seaweed as a functional ingredient in cracker formulations, affecting both textural properties and sensory experiences. It also highlights the importance of pre‐processing treatments in modulating these effects, providing valuable insights for the development of seaweed‐fortified food products with enhanced nutritional value and consumer acceptability.

## Graphical abstract

The graphical abstract presents the concept of a seaweed‐based cracker, showcasing its development and innovative use. It illustrates the key steps in the creation process of fortifying wheat crackers with seaweed species, highlighting the product's textural and consumer evaluations.

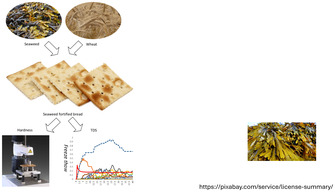

## 1 Introduction

As the world's population is increasing, there is a demand for food to feed the human population. To meet the increasing food requirements, alternative solutions need to be sought. Seaweeds are a useful ingredient for sustainability and globally 145 species of seaweed are consumed as food (Buckley et al. 2023). Seaweeds or edible algae are rich in micronutrients, found on the coast of many countries, are valued as marine plants (Keyimu 2013), and according to Bequette and France (1997) there are ~45,000 species of marine macroalgae or seaweed belonging to three distinct groups: Brown (*Phaeophyceae*), Green (*Chloropgyceae*), and Red (*Rhodophyceae*).

In Europe, seaweeds have been consumed for decades, but their flavors and textures often carry a negative perception among consumers (Mendis and Kim 2011). Seaweeds are rich in a wide variety of bioactive components, contributing to many health benefits, and accordingly can be classified as a source of functional food ingredients (Cox and Abu‐Ghannam 2013). Nevertheless, seaweeds remain largely unexploited as a food source in the Western diet and are principally used to provide extracts such as agar, carrageenan, and alginate. Seaweeds have potential as a food source, as it contains a source of nutrients, including many essential vitamins (vitamin K, B vitamins) and minerals (Zinc and Iron) (Roohinejad et al. 2017; Cox and Abu‐Ghannam 2013; Amorim, Lage‐Yusty, and López‐Hernández 2012; Kadam and Prabhasankar 2010; Tibbetts, Milley, and Lall 2016) and a significant source of fiber, carbohydrates, protein, and essential fatty acids (MacArtain et al. 2007).

Considering the functional attributes of seaweeds, they have extensive uses and potential incorporation into many food products (Roohinejad et al. 2017). With the application of seaweeds and seaweed extracts added to food products (fortification), it could increase the nutritional value, and improve the texture and sensory profiles of foods. The processing of seaweeds, particularly through heat treatments, has emerged as a promising avenue to extract and enhance the bioactive compounds.

Enzymatic hydrolysis and thermal treatments including steam blanching and microwave‐assisted extraction have been explored to release bioactive compounds from seaweed matrices (Dumay and Morançais 2015) aiming to improve the extraction efficiency of valuable compounds, such as proteins, polysaccharides, and polyphenols. The processing of seaweeds through techniques such as pasteurization and blanching plays a crucial role in enhancing their safety, shelf life, and overall palatability. Pasteurization is particularly relevant for seaweeds destined for consumption in raw or minimally processed forms. Studies have shown that pasteurization effectively reduces the microbial load in seaweeds, ensuring the safety of the final products (Munir et al. 2019). The nutritional impact of pasteurization on seaweeds is a critical aspect to consider. While these heat treatments can result in minimal losses of certain heat‐sensitive nutrients, such as vitamins and antioxidants, the overall nutritional profile of seaweeds remains robust (Rajauria, Foley, and Abu‐Ghannam 2016).

The incorporation of heat‐processed seaweeds into food products has gained momentum, driven by consumer demand for novel, nutritious, and sustainable alternatives. Studies have explored the addition of seaweed extracts to enhance the physicochemical and sensory characteristics of meat products. This not only showcases the versatility of seaweeds in various culinary applications but also underscores their potential as functional ingredients.

Sensory analysis and palatability were investigated in a study by Blouin et al. (2006), in which children and adults were the participants in a trial comparing *Porphyra* species in crackers and popcorn. Children found the popcorn samples higher in acceptability although the adults also found it palatable with marginally rating the crackers of higher acceptability. A noticeable flavor was detected and accepted on both *Porphyra* species and food products. This could explain the consumer's previous experience with a similar product to be texturally accepted. In a different study, Wilkin et al. (2021) optimized the concentration of two brown seaweeds in crackers and assessed consumer acceptability using temporal dominance of sensation (TDS), finding that at low concentrations both species expressed similar attributes but at higher concentrations less desirable seaweed attributes overpowered the crackers.

The nutritional profile and potential health benefits of a new food are insufficient alone to improve nutritional outcomes. The consumer experience needs to be positive, especially the sensory experience including texture, taste, smell, flavor, and appearance attributes contributing to the overall palatability of the food. More recently the application of technology has allowed the use of technology through check‐all that apply (CATA) and TDS.

## 2 Materials and Methods

### 2.1 Materials

Seaweed species *Laminaria digitata* (oarweed), *Alaria esculenta* (winged kelp), and *Saccharina latissima* (sugar kelp) were supplied by Biomara Ltd. (Edinburgh, UK). Seaweeds were dried using indirect heating, temperatures varied between 35°C and 55°C depending on the stage in the drying cycle, before milling. The seaweeds were milled using a centrifugal mill fitted with a stainless‐steel ring sieve with 1.0 mm trapezoid holes (Ultra Centrifugal Mill ZM 200, Retsch, Germany). The samples were stored in a cool, dark place before use.

Plain flour, rapeseed oil, white granulated sugar, and cooking salt were purchased from a local supermarket.

High‐performance chromatography (HPLC) grade methanol was purchased from Thermo Fisher Scientific (UK), cycloleucine (97%) was purchased from Sigma Aldrich (Gillingham, UK). N‐methyl‐N‐(trimethylsilyl) trifluoroacetamide (MSTFA) (100%) was purchased from Fluorochem (Hadfield, UK).

### 2.2 Seaweed Treatments

Seaweed was rehydrated at a ratio of 1:2 w/w (seaweed:water) and sealed in heat‐sealed bags. Samples were subjected to retort, a freeze–thaw cycle, or no treatment (control). Retorted samples were heated to 121°C for 30 min (FT19‐A Portable autoclave, Dixons). Freeze–thaw samples were frozen for 24 h at −18°C, samples were then thawed at ambient temperature and refrozen until required.

### 2.3 Cracker Production

The base cracker recipe used the following ingredients: flour (65.0 g), water (42.75 g), rapeseed oil (5.0 g), and sugar (2.5 g). Seaweed was incorporated into a standardized cracker dough at concentrations of 0%, 5%, 10%, and 15% dw, substituted for flour. The water added to the dough was reduced accounting for the “rehydrated” water added with seaweed. The control had 5% less water than the seaweed substituted crackers to allow manipulation of the dough.

The cracker dough was mixed by hand, rolled out using a pasta maker to a thickness of 2 mm. The dough was cut into 28 mm squares using a grid cutter, placed on a baking sheet in the center of the oven at 180°C for 8 min. Once cooked the crackers were transferred to a dehydrator (4900B 9 tray, Excalibur) set at 74°C for 16 h. Crackers were then cooled and placed in heat‐sealed bags.

### 2.4 Moisture Content

The moisture content of the crackers was determined by oven drying at 110°C for 24 h in triplicate. The moisture content was calculated using equation (Equation 1).

(1) $$\%\text{Moisture}=\left(\frac{m_{\mathrm{wet}}-m_{\mathrm{dry}}}{m_{\mathrm{dry}}}\right)\times 100$$

where *m*_wet_ and *m*_dry_ were the weight of the sample before and after the oven drying.

### 2.5 Bake Loss and Shrinkage

The mass of crackers was weighed pre and post‐cooking/dehydrating to calculate bake loss. The size of the crackers was measured using a standard ruler measuring *x*×*y* in mm, pre, and post‐cooking/dehydrating to calculate shrinkage.

### 2.6 TDS Analysis

Previous studies suggest that the concentration of seaweed has an overpowering effect on cracker TDS (Wilkin et al. 2021), therefore only 15% samples were used in this analysis. Panelists were recruited from within Abertay University. In total, 12 semi‐trained panelists (six females; six males) participated. The following inclusion criteria were used: an interest in healthy eating, aged between 20 and 60 years old, had good general health, non‐smokers, could distinguish between sensory attributes from a questionnaire, fluent in English and used computers frequently. TDS analysis was conducted at Abertay University's Food Sensory Consumer Labs (ISO8589:2007) using Compusense (Ontario, Canada). To help guide the panelists in understanding the testing phase a decoy sample was prepared to provide them with experience of a TDS sample, these results were not recorded. Panelists were then provided with a maximum of eight crackers per sitting (with a gap in testing after four samples), samples were run in triplicate, each Panelist came back for repeated sessions four times, with a total of 30 crackers tested by each participant.

### 2.7 Textural Analysis

Cracker samples underwent texture analysis using the TA.XTX2 texture analyzer (Stable Microsystems, Surrey, UK). All analyses were carried out at room temperature, within 1 h of removal from the dehydrator. The sample was analyzed (*n* = 6), using “a ¼” ball probe, with the sample placed on the support rig before being compressed using a 5 kg load cell. Breakpoint (N) was recorded using the force‐in compression tests. The analyzer was set to a return‐to‐start cycle with calibrated probe height, a pre‐test speed of 10 mm/s and a test speed of 20 mm/s, and a post‐test speed of 10 mm/s was used. Trigger force was 5 g, and the probe distance was set at 50 mm (Abdel‐Samie et al. 2010).

### 2.8 Statistical Analysis

All experiments were conducted in triplicate (unless specified), and the results were analyzed with a three‐way analysis of variance (ANOVA) test at the 95% significant level (*p* < 0.05) using SPSS (version 29). Potential statistical outliers were investigated using both Grubbs' test and Dixon's test, with the critical values for *p* = 0.05 used.

## 3 Results and Discussion

### 3.1 Cracker Physical Properties

Crackers were assessed for moisture content, shrinkage, and bake loss before baking and following both baking and dehydrating.

**FIGURE 1:**
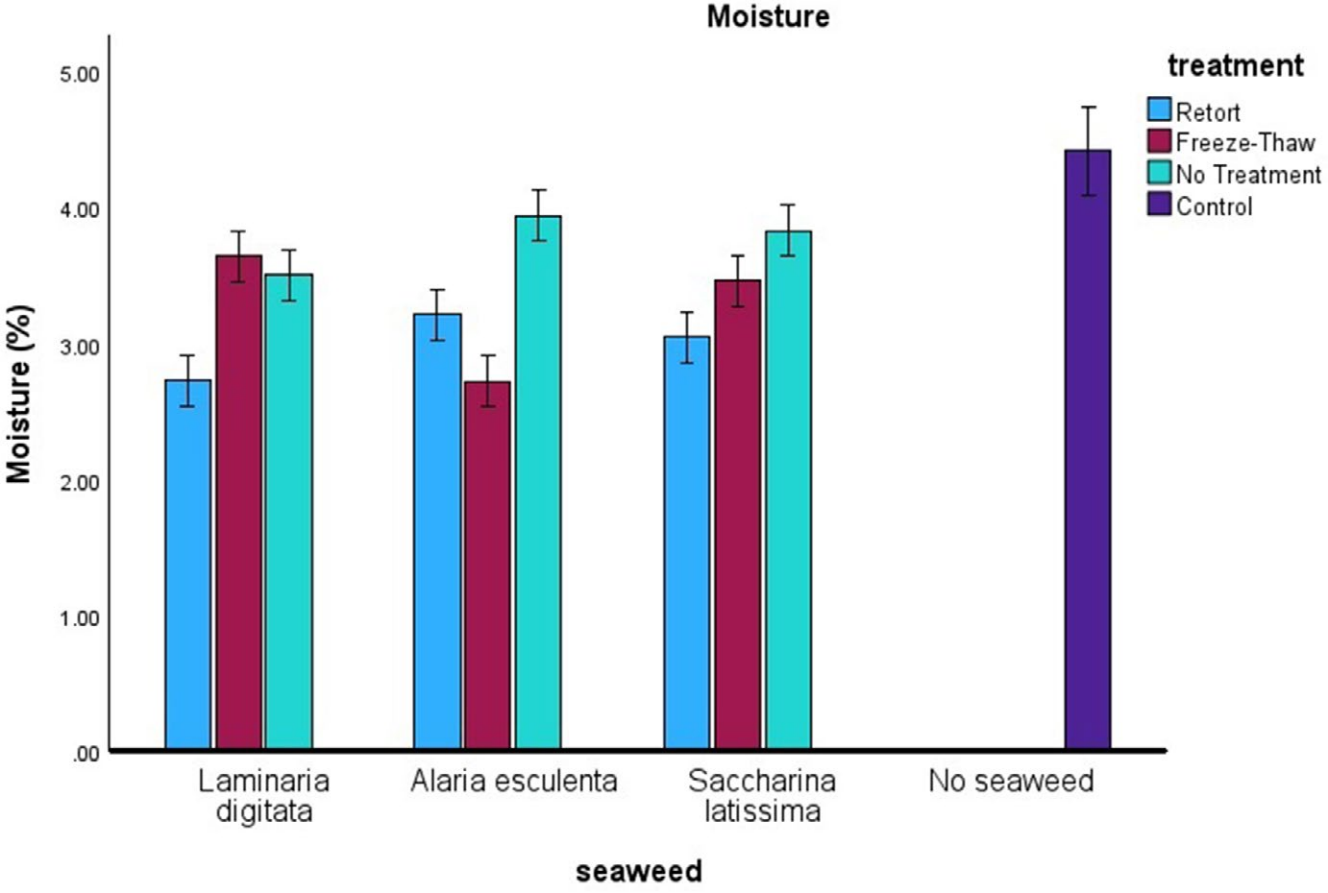
Moisture content (%) of crackers is shown against the species of seaweed on the *x*‐axis, with the different treatments represented by colors: Light blue for retort, red for freeze–thaw, and light green for no treatment. The control is indicated in dark blue.

Results show that cracker moisture content was predominantly affected by the seaweed thermal treatment (Figure 1). A univariant between subjects' effects test was conducted and identified two‐way interactions of seaweed versus treatment, seaweed versus incorporation, and treatment versus incorporation as all being significant (*n* = 84, *F* = 18.836, *p* ≤ 0.001, *F* = 5.799, *p* ≤ 0.001 and *F* = 5.221, *p* = 0.001, respectively). When combining seaweed species, treatment and incorporation rate, an increased significance compared to two‐way interactions was observed (*n* = 84, *F* = 8.739, *p* ≤ 0.001). A post hoc Tukey test showed that retort treatments and freeze–thaw treatments had a significantly different moisture content compared to untreated seaweed crackers. In this study the incorporation rate had no significant effect (*p* = 0.05) on the moisture content except the control (0%), indicating once the product was fortified, the effect on moisture loss was minimal. The addition of seaweed resulted in a drier cracker (lower moisture content), this could be caused by the addition of seaweed weakening the glutenous bond allowing moisture to escape the product more easily on cooking, however, this was contrary to the review findings of Kumar et al. (2018) and Ainsa et al. (2022).

**FIGURE 2:**
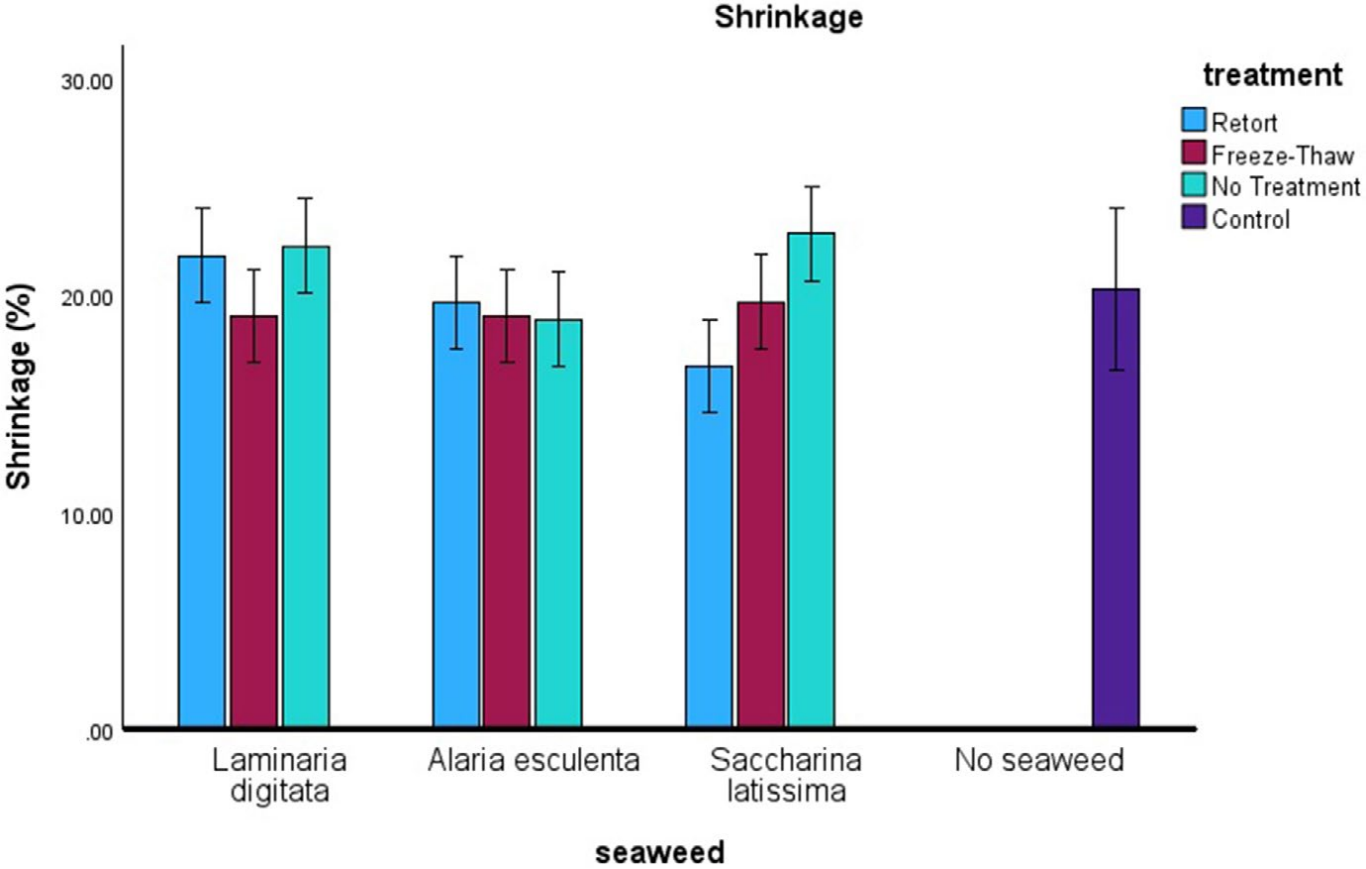
Shrinkage (%) of crackers is shown against the species of seaweed on the *x*‐axis, with the different treatments represented by colors: Light blue for retort, red for freeze–thaw, and light green for no treatment. The control is indicated in dark blue.

Cracker shrinkage (Figure 2) was predominantly affected by the sample treatment (*n* = 84, *F* = 3.448, *p* = 0.039), a slight increase in significance was found for the two‐way interaction for seaweed species and treatment (*n* = 84, *F* = 3.642, *p* = 0.010). There were no statistically significant two‐way or three‐way interactions within shrinkage and thermal treatments or incorporation rates (*p* > 0.05). Although the seaweed crackers showed a difference in moisture content, shrinkage was not affected, this could be attributed to the seaweed alginates acting similarly to gluten. The gluten network in the control cracker provided structure for the cracker, and thus very little shrinkage occurs, whereas gluten was reduced in the crackers via the substitution of flour with milled seaweed. During the production of the crackers, the dough was kneaded, and this action alongside the wetting of the dough mixture allowed for the alginates to come into suspension within the food matrix. Once baked and moisture removed, the alginates solidified and provided a similar structure to gluten in this trial. Previous studies have shown that potato starch depleted the height and structure of breads when substituted with wheat flour, however, the addition of sodium alginate helped keep the structure of these breads (Liu 2017).

**FIGURE 3:**
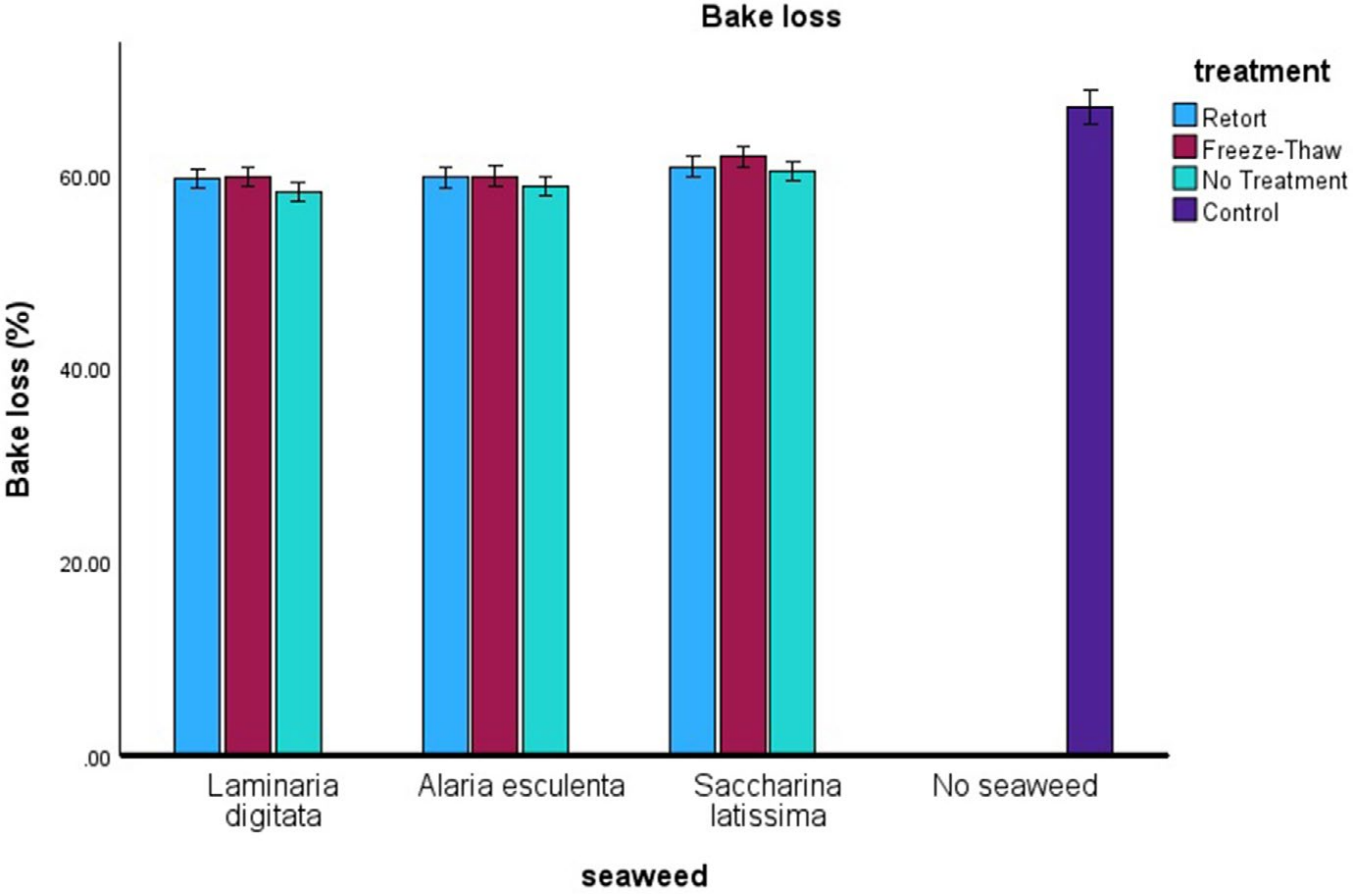
Bake loss (%) of crackers is shown against the species of seaweed on the *x*‐axis, with the different treatments represented by colors: Light blue for retort, red for freeze–thaw, and light green for no treatment. The control is indicated in dark blue.

For bake loss (Figure 3) the single factor of seaweed species was significant (*n* = 84, *F* = 10.799, *p* ≤ 0.001), there are no significant two‐way interactions, but considering three‐way interactions of species, treatment, and incorporation rate were significant (*n* = 84, *F* = 2.667, *p* = 0.015). *S. latissima* was significantly different in bake loss compared to both *L. digitata* and *A. esculenta* and the control (absence of seaweed). Interestingly the addition of seaweed to crackers resulted in a lower bake loss (contrary to moisture content), *S. latissimi* had a higher bake loss compared to the other species (*L. digitata* and *A. esculenta* ). Previous reports show that *L. digitata* when compared with *S. latissima* showed higher density in cellular walls before processing and showed a greater release of structural polysaccharides (Souto‐Prieto et al. 2024). The authors suggested that these two seaweeds' cellular structural organization might be different and that this affected their flow during rheological trials. More work is required to fully understand how seaweeds affect the physical properties of food products when used to fortify and how these can alter the food products, and once understood optimized to match consumer needs.

### 3.2 Texture Analysis

**FIGURE 4:**
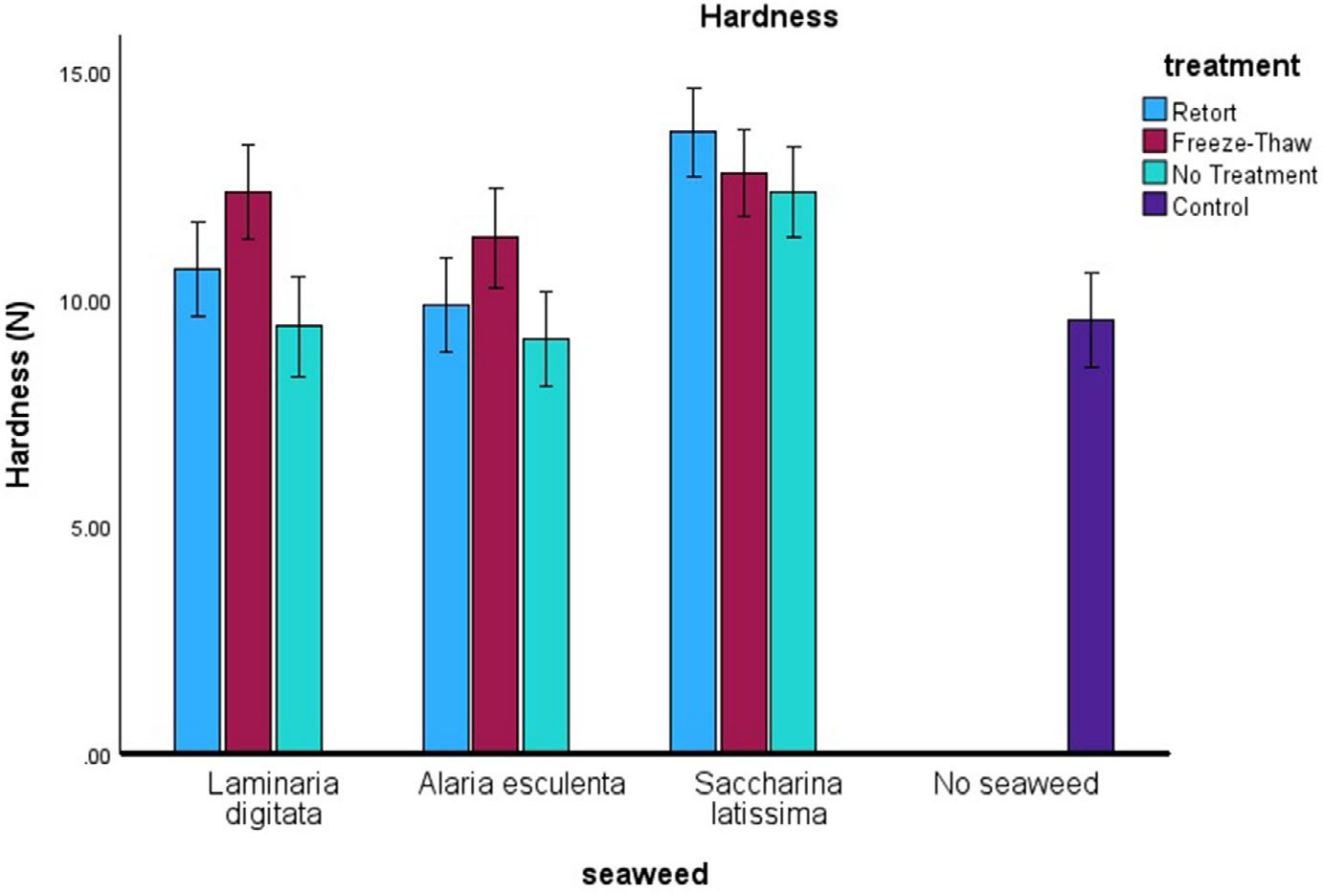
Hardness (N) of crackers is shown against the species of seaweed on the *x*‐axis, with the different treatments represented by colors: Light blue for retort, red for freeze–thaw, and light green for no treatment. The control is indicated in dark blue.

For hardness, individual factors of both seaweed species and the treatment (Figure 4), all two‐way interactions seaweed versus treatment, seaweed versus incorporation, and treatment versus incorporation (*n* = 139, *F* = 2.549, *p* = 0.043, *F* = 3.346, *p* = 0.013, *F* = 8.777, *p* ≤ 0.001 respectively) and the three‐way interaction of seaweed, treatment, and incorporation were significant (*n* = 139, *F* = 2.257, *p* = 0.028). A post hoc Tukey analysis identified *S. latissima* as significantly different from both *L. digitata* , *A. esculenta and* the control (no seaweed added). Freeze–thaw treatment was significantly different than no treatment and the control (no seaweed added), producing crackers that were significantly harder for all species of seaweed. The increased hardness observed for crackers fortified with seaweed was likely due to the increased fiber in the cracker. However, the freeze–thaw process changed the structure of the polysaccharides attached to the cellular structure of the milled seaweed and allowing it to seep out during cracker production, thus making this a harder cracker. Recent studies have correlated well with our study, where observed differences were seen with the addition of seaweed powders to cracker mixes (Aganduk 2023). The texture analysis showed that seaweed species was a controlling factor, but thermal treatments affected the overall texture of the product. Therefore when the food industry is looking to extend the shelf life of seaweeds through thermal treatments better care should be taken to fully understand the role of the thermal process on the ingredient interactions.

### 3.3 TDS

**FIGURE 5:**
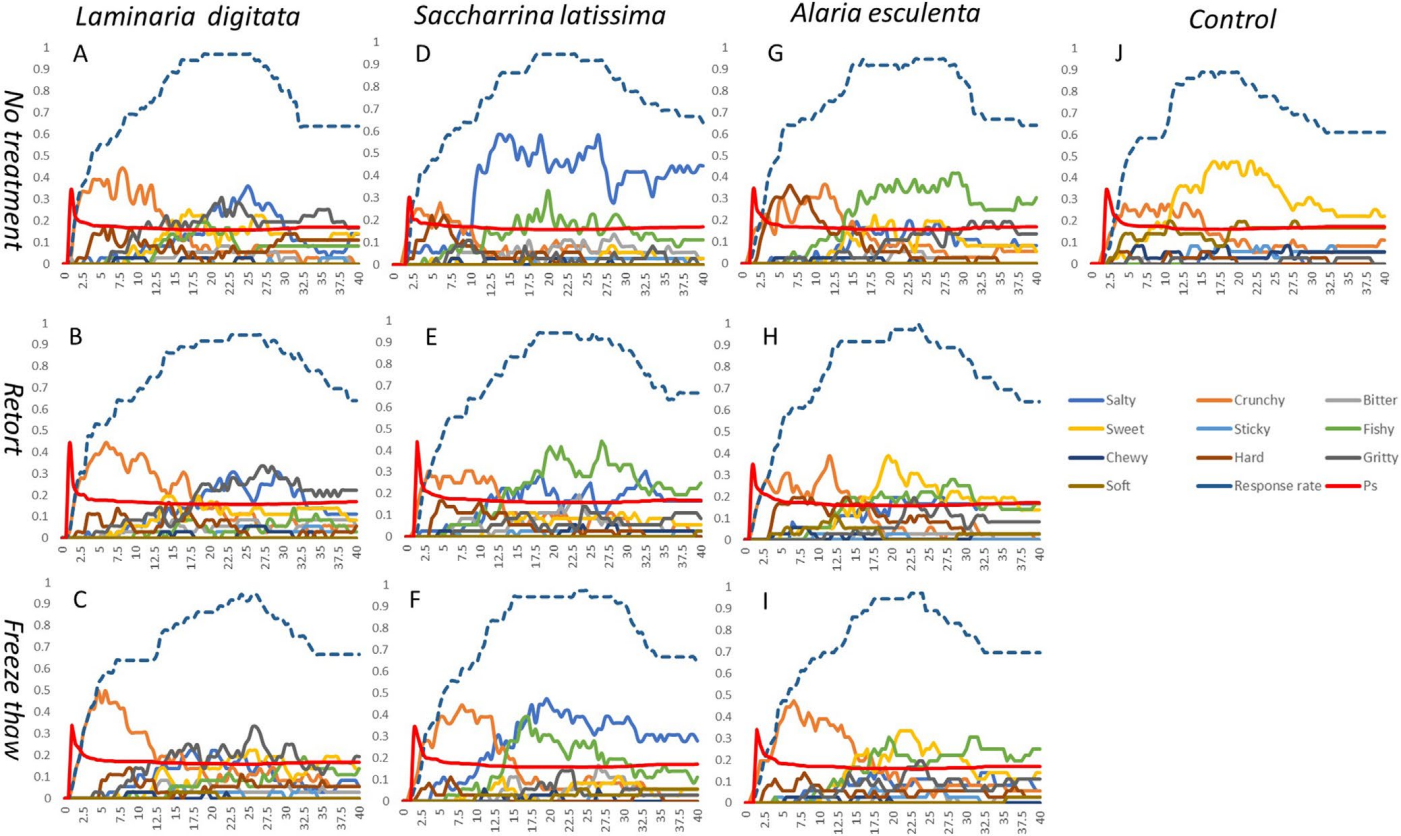
Temporal dominance of sensations (TDS) is presented for each species of seaweed ( *Laminaria digitata* (A–C), *Saccharina latissima* (D–F), and *Alaria esculenta* (G–I)) under the no treatment condition (A, D, G), retort (B, E, H), and freeze–thaw (C, F, I), as well as the control (J), which contained no seaweed. The *y*‐axis represents the dominance of the attribute, while the *x*‐axis shows time in seconds. The response rate indicates the number of times an attribute was selected by the cohort of consumers. The Ps line represents statistical significance, and attributes crossing this line are reported.

Figure 5 shows the TDS for each of the 15% species or treatment of crackers. The control cracker (Figure 5J) was made using no seaweed and the most dominant attribute was the crunchy attribute within the first 12 s of eating, followed by an overpowering sweetness for the rest of the eating process. *L. digitata* species crackers showed the most dominant sensation as crunchy within the first 15 s of eating. Within the No treatment *L. digitata* (Figure 5A) cracker, a small 5 s of sweetness was most dominant, which was not seen within the other treatments for this species. Freeze–thaw treatment for *L. digitata* (Figure 5C) showed mainly a gritty sensation which lingered well after the swallow of the product (around 20 s) of chewing. Retorted *L. digitata* (Figure 5B) showed similar trends to Freeze Thraw *L. digitata* , but more pronounced in terms of dominance, combined with the salty flavor. However, the retorted cracker (Figure 5B) had a longer crunch in terms of time being dominant than freeze–thawed (Figure 5C). *S. latissima* 15% no treatment (Figure 5D) cracker had a dominant sensation of crunchiness for the first 10 s of the product which was taken over by a high saltness. Whereas the retorted *S. latissima* (Figure 5E) had a slight increase in crunchy attribute from 10 to 12.5 s from no treatment. Interestingly unlike the overpowering attribute of saltness for the No Treatment (Figure 5D), the retorted cracker (Figure 5E) had an overriding fishy flavor coming through. The Freeze–thaw *S. latissima* (Figure 5F) had a longer and more pronounced crunchy dominance when compared with the No Treatment (Figure 5D) but correlated well with the No Treatment in terms of salty flavor.

*A. esculenta* 15% No treatment (Figure 5G) cracker had a dissimilar TDS profile than *L. digitata* and *S. latissima* (Figure 5A,D), where crunchy was not the first dominant attribute, but hardness was. The hard attribute was then replaced as most dominant by the crunchy attribute, briefly, before the fishy flavor became the most dominant attribute for the rest of the eating process. Interestingly the Retort *A. esculenta* (Figure 5H) cracker reduced the hard attribute through increasing the crunchy attribute. Then sweetness took over from 15 s through to 27.5 s, where the fishy back notes were detected. Retorted seemed to have made the *A. esculenta* cracker more crunchy and less fishy. Freeze–thaw (Figure 5I) also reduced the hard attribute and crunchy was the most dominant attribute for the first 15 s, where fishy and sweet alternated between being the most dominant attribute through the rest of the eating process.

Seaweed species had a real difference in their eating processes in terms of most dominant attributes, and the retorted seaweeds showed in each species a difference in the texture of the product, by prolonging the crunchiness within the first 20 s of eating. Retort and Freeze–thaw both showed that some of the more unpleasant flavors (fishy) could be reduced through this type of processing before incorporating into a food product. This TDS data correlated nicely with the texture analysis data, where the treatments of retorting and Freeze–thaw increased the hardness of the products compared to no treatments. The change of a few Newtons in terms of hardness could be increasing the bite of the crackers and therefore when mastication occurred and the product was being rolled around the mouth forming a bolus, the crunchy attribute was deemed higher for the consumer.

Another reason could be that the crackers made with the retorted and freeze–thawed seaweeds had lower moisture content and a higher bake loss than those with seaweeds of other treatment, where the seaweed structure was being destroyed during these processes and was reducing the water holding capacity of the seaweed inclusions within the product. Ge et al. (2002) suggested that heat treatments above 118°C caused damage to the cellular structure of these marine crops (Mateluna et al. 2020). The damage in microstructure of the retorted seaweeds could explain a higher bake loss and a lower moisture content of the crackers which in turns leading to the crunchy attribute in the TDS and the harder values for texture.

## 4 Conclusion

Thermal treatments on the seaweeds had an impact on the overall texture and consumer appeal on the seaweed cracker. Interestingly both retorting and freeze–thaw processes affected the texture of crackers, compared with no treatments, but the inclusion of seaweed still dominated this. Further work is required to understand the mechanisms involved in the seaweed products, which could explain the changes in structure as well as the changes to the cracker consumption. The interplay between species of seaweeds, thermal treatments and inclusion rates still requires further development to fully understand the changes each of these have in terms of product development.

## Conflicts of Interest

The authors declare no conflicts of interest.

## Data Availability

The data supporting this study's findings are openly available in the Figshare Repository at https://doi.org/10.6084/m9.figshare.26303890.v1.

## References

[fsn34710-bib-0001] Abdel‐Samie, M. A.‐S. , J. Wan , W. Huang , O. K. Chung , and B. Xu . 2010. “Effects of Cumin and Ginger as Antioxidants on Dough Mixing Properties and Cookie Quality.” Cereal Chemistry 87: 454–460.

[fsn34710-bib-0002] Aganduk, A. , P. Matanjun , T. S. Tan , and B. H. Khor . 2023. “Proximate and Physical Analyses of Crackers Incorporated With Red Seaweed, *Kappaphycus Alvarezii* .” Journal of Applied Phycology 36: 1–7.

[fsn34710-bib-0004] Ainsa, A. , A. Honrado , P. Marquina , J. A. Beltrán , and J. Calanche . 2022. “Influence of Seaweeds on the Quality of Pasta as a Plant‐Based Innovative Food.” Food 11, no. 16: 2525. 10.3390/foods11162525 PMC9407415 36010525

[fsn34710-bib-0005] Amorim, K. , M.‐A. Lage‐Yusty , and J. López‐Hernández . 2012. “Changes in Bioactive Compounds Content and Antioxidant Activity of Seaweed After Cooking Processing.” CyTA Journal of Food 10, no. 4: 321–324.

[fsn34710-bib-0006] Bequette and France . 1997. “Seaweed at Your Service.” In The UNESCO Courier: a Window Open on the World, 40–42. France: United Nations Education, Scientific and Cultural Organisation.

[fsn34710-bib-0007] Blouin, N. , B. L. Calder , B. Perkins , and S. H. Brawley . 2006. “Sensory and Fatty Acid Analyses of Two Atlantic Species of Porphyra (Rhodophyta).” Journal of Applied Phycology 18: 79–85.

[fsn34710-bib-0008] Buckley, S. , K. Hardy , F. Hallgren , et al. 2023. “Human Consumption of Seaweed and Freshwater Aquatic Plants in Ancient Europe.” Nature Communications 14: 6192. 10.1038/s41467-023-41671-2 PMC10582258 37848451

[fsn34710-bib-0009] Cox, S. , and N. Abu‐Ghannam . 2013. “Enhancement of the Phytochemical and Fibre Content of Beef Patties With *Himanthalia elongata* Seaweed.” International Journal of Food Science & Technology 48, no. 11: 2239–2249.

[fsn34710-bib-0010] Dumay, J. , and M. Morançais . 2015. “Use of a Two‐Step Extraction Method to Improve Lipid Extraction From Edible Seaweeds.” Food Chemistry 171: 324–331.

[fsn34710-bib-0011] Ge, M. , J. Shen , C. Liu , W. Xia , and Y. Xu . 2002. “Effect of Acidification and Thermal Treatment on Quality Characteristics of High‐Moisture Laver (*Porphyra spp*.).” Journal of Food Processing and Preservation 46: e16762.

[fsn34710-bib-0012] Kadam, S. U. , and P. Prabhasankar . 2010. “Marine Foods as Functional Ingredients in Bakery and Pasta Products.” International Food Research Journal 43, no. 8: 1975–1980.

[fsn34710-bib-0013] Keyimu, X. G. 2013. “The Effects of Using Seaweed on the Quality of Asian Noodles.” Journal of Food Processing & Technology 4: 1–4.

[fsn34710-bib-0014] Kumar, A. , E. Krishnamoorthy , H. M. Devi , et al. 2018. “Influence of Sea Grapes ( *Caulerpa racemosa* ) Supplementation on Physical, Functional, and Anti‐Oxidant Properties of Semi‐Sweet Biscuits.” Journal of Applied Phycology 30: 1393–1403.

[fsn34710-bib-0015] Liu, X. , T. Mu , K. D. Yamul , et al. 2017. “Evaluation of Different Hydrocolloids to Improve Dough Rheological Properties and Bread Quality of Potato–Wheat Flour.” Journal of Food Science and Technology 54: 1597–1607. 28559619 10.1007/s13197-017-2591-y PMC5430192

[fsn34710-bib-0016] MacArtain, P. , C. I. R. Gill , M. Brooks , R. Campbell , and I. R. Rowland . 2007. “Nutritional Value of Edible Seaweeds.” Nutrition Reviews 65, no. 12: 535–543. 18236692 10.1301/nr.2007.dec.535-543

[fsn34710-bib-0017] Mateluna, C. , V. Figueroa , J. Ortiz , and J. M. Aguilera . 2020. “Effect of Processing on Texture and Microstructure of the Seaweed Durvillaea Antarctica.” Journal of Applied Phycology 32: 4211–4219.

[fsn34710-bib-0018] Mendis, E. , and S.‐K. Kim . 2011. “Advances in Food and Nutrition Research.” In Present and Future Prospects of Seaweeds in Developing Functional Foods, edited by S. Kim , 1–15. s.l. London: Academic Press. 10.1016/B978-0-12-387669-0.00001-6 22054934

[fsn34710-bib-0019] Munir, M. T. , S. A. Siddiqui , M. M. Rahman , and S. Ahmad . 2019. “Effect of Different Pasteurization Methods on the Quality of Seaweed (Gracilaria Changii) Beverages.” LWT 101: 104–109.

[fsn34710-bib-0021] Rajauria, G. , B. Foley , and N. Abu‐Ghannam . 2016. “Identification and Characterization of Phenolic Antioxidant Compounds From Brown Irish Seaweed *Himanthalia elongata* Using LC‐DAD–ESI‐MS/MS.” Innovative Food Science & Emerging Technologies 37: 261–268.

[fsn34710-bib-0022] Roohinejad, S. , M. Koubaa , F. J. Barba , S. Saljoughian , M. Amid , and R. Greiner . 2017. “Application of Seaweeds to Develop New Food Products With Enhanced Shelf‐Life, Quality and Health‐Related Beneficial Properties.” Food Research International 99, no. 3: 1066–1083. 28865618 10.1016/j.foodres.2016.08.016

[fsn34710-bib-0023] Souto‐Prieto, A. , M. Martinez‐Sanz , T. Ferreiro , et al. 2024. “Insights Into the Structuring Ability of Two Brown Seaweeds (*Laminaria Digitata* and *Saccharina Latissima*) for Applications as Natural Texturisers.” Algal Research 80: 103548.

[fsn34710-bib-0024] Tibbetts, S. M. , J. E. Milley , and S. P. Lall . 2016. “Nutritional Quality of Some Wild and Cultivated Seaweeds: Nutrient Composition, Total Phenolic Content and In Vitro Digestibility.” Journal of Applied Phycology 28: 3575–3585.

[fsn34710-bib-0026] Wilkin, J. D. , K. Ross , T. Alric , M. Hooper , J. V. Grigor , and B. S. Chu . 2021. “Optimisation of Concentration of Undaria Pinnarifida (Wakame) and Himathalia Elongate (Sea Spaghetti) Varieties to Effect Digestibility, Texture and Consumer Attribute Preference.” Journal of Aquatic Food Product Technology 30, no. 8: 932–943.

